# *In vitro* evaluation of novel antimicrobial coatings for surgical sutures using octenidine

**DOI:** 10.1186/s12866-015-0523-4

**Published:** 2015-09-24

**Authors:** A. Obermeier, J. Schneider, P. Föhr, S. Wehner, K.-D. Kühn, A. Stemberger, M. Schieker, R. Burgkart

**Affiliations:** Klinikum rechts der Isar, Technische Universität München, Klinik für Orthopädie und Sportorthopädie, Ismaninger Str. 22, 81675 Munich, Germany; Klinikum rechts der Isar, Technische Universität München, Institut für Mikrobiologie, Immunologie und Hygiene, Trogerstr. 30, 81675 Munich, Germany; Klinikum der Universität München, Klinik für Chirurgie, Experimentelle Chirurgie und Regenerative Medizin, Nußbaumstr. 20, 80336 Munich, Germany; Universitätsklinik für Orthopädie und Orthopädische Chirurgie, Medizinische Universität, Auenbruggerplatz 5, 8036 Graz, Austria

**Keywords:** Surgical sutures, Antimicrobial, Coating, Octenidine, Slow-release, Fatty acid, Staphylococcus aureus, Surgical site infection, Biocompatibility, *In vitro*

## Abstract

**Background:**

Sutures colonized by bacteria represent a challenge in surgery due to their potential to cause surgical site infections. In order to reduce these type of infections antimicrobially coated surgical sutures are currently under development. In this study, we investigated the antimicrobial drug octenidine as a coating agent for surgical sutures. To achieve high antimicrobial efficacy and required biocompatibility for medical devices, we focused on optimizing octenidine coatings based on fatty acids. For this purpose, antimicrobial sutures were prepared with either octenidine-laurate or octenidine-palmitate at 11, 22, and 33 μg/cm drug concentration normalized per length of sutures. Octenidine containing sutures were compared to the commercial triclosan-coated suture Vicryl® Plus. The release of octenidine into aqueous solution was analyzed and long-term antimicrobial efficacy was assessed via agar diffusion tests using *Staphylococcus aureus*. For determining biocompatibility, cytotoxicity assays (WST-1) were performed using L-929 mouse fibroblasts.

**Results:**

In a 7 days elution experiment, octenidine-palmitate coated sutures demonstrated much slower drug release (11 μg/cm: 7 %; 22 μg/cm: 5 %; 33 μg/cm: 33 %) than octenidine-laurate sutures (11 μg/cm: 82 %; 22 μg/cm: 88 %; 33 μg/cm: 87 %). Furthermore sutures at 11 μg/cm drug content were associated with acceptable cytotoxicity according to ISO 10993–5 standard and showed, similar to Vicryl® Plus, relevant efficacy to inhibit surrounding bacterial growth for up to 9 days.

**Conclusions:**

Octenidine coated sutures with a concentration of 11 μg/cm revealed high antimicrobial efficacy and biocompatibility. Due to their delayed release, palmitate carriers should be preferred. Such coatings are candidates for clinical testing in regard to their safety and efficacy.

## Background

Surgical site infection (SSI) is a common complication after surgical intervention and incidence for SSI can be as high as 25 %, depending on the anatomical location of the surgical site [1–5]. The onset of SSI has been associated with a variety of factors, including surgical sutures [6]. The property of surgical suture for adhering bacteria promotes the occurrence of such infections [7]. The use of antimicrobially coated sutures poses one possible approach to prevent or reduce suture-associated infections. At present, the only commercially available antimicrobial sutures are coated with triclosan, such as the resorbable multifil Vicryl® Plus [8]. Several *in vitro* studies reported that triclosan-incorporated sutures showed high antimicrobial activity against a broad spectrum of pathogens [9–11]. Dependent on the surgery site, the clinical benefit of such coated sutures varies. On the one hand, no benefit was reported in studies for appendicitis, breast cancer or colorectal surgery [3, 12–14]. In contrast, the use of antimicrobial coated sutures in sternum surgery [15], abdominal wall closure [16, 17] and cerebrospinal fluid shunting procedures [18] was associated with significantly lower wound infection rates. Moreover, a recent meta-analysis showed a significant benefit of triclosan coated sutures to prevent surgical site infection [19]. However, due to its wide use in health-care, household and cosmetic items triclosan resistance is frequently reported for *S. aureus* a common pathogen in wound infections [20]. Furthermore the promotion of multi drug resistances in the presence of triclosan via increased activity of efflux pump system is a major concern [21]. Screening tests performed between 2003 and 2004 already demonstrated detection of triclosan in the urine of 74.6 % of US citizens [22, 23]. Therefore, the development of sutures with different antimicrobial drugs is urgently required.

In a previous study, we established a coating process to render surgical sutures antiseptic, while meeting the European Pharmacopoeia requirements for the material strength of surgical sutures [24, 25]. We showed that sutures coated with the antimicrobial drug chlorhexidine pose a highly efficient alternative with low cytotoxicity to the established antimicrobial sutures using triclosan, such as Vicryl® Plus. Furthermore we demonstrated a strongly dose dependent efficacy and biocompatibility. However, the antimicrobial effect lasted up to 4 days and biocompatibility was also limited. Therefore, we chose the antimicrobial drug octenidine for further development of suture coatings. Octenidine is well established in skin and wound antiseptic solutions, and even recommended as potential alternative in case of triclosan resistance [26]. Further, this antiseptic drug has a broad-spectrum activity, including common pathogens of wound infections such as multiresistant bacteria [27]. In addition, the lower solubility in aqueous medium may result in slower drug release, with longer efficacy against pathogens and lower cytotoxicity compared to chlorhexidine coatings.

The aim of the present study was the development of antimicrobial octenidine containing formulations based on fatty acids. We focused on optimizing drug concentrations in order to achieve improved long-term antimicrobial efficacy and biocompatibility concerning resorbable sutures. Both antimicrobial efficacy over several days, as well as cytotoxicity of novel coated octenidine containing sutures were compared to commercially available plain polyglycolic acid (PGA) and triclosan containing sutures like Vicryl® Plus.

## Methods

### Surgical sutures

Sutures in our study had diameters according to United States Pharmacopeia (USP 1). The used suture consists of polyglycolic acid (PGA, Gunze Ltd., Japan), free of fatty acids to avoid sewing effect. Our investigations were compared with reference sutures (PGA Resorba®, Resorba Medical GmbH, Germany; Vicryl® and Vicryl® Plus, Ethicon GmbH, Germany).

### Antimicrobial coating solutions

Antimicrobial coating solutions were prepared as follows: Either palmitic or lauric acid together with octenidine were dissolved in 99.8 % ethanol (Carl Roth GmbH, Germany). The solutions contained 395.0 mg of both components fatty acid and octenidine in 7.9 g (10.0 ml) ethanol, which corresponds to a mass content of 5 % (w/w). Under aseptic conditions, the solutions had to be homogenized and filtered (minisart, sartorius AG, Germany, pore size 0.2 μm). Two types of coating were produced: Octenidine dihydrochloride (Dishman Pharmaceuticals & Chemicals Ltd., India) in lauric acid (**OL**) and Octenidine dihydrochloride in palmitic acid **(OP)**. Both for OL and OP, three different antiseptic drug concentrations inside coating solutions were chosen: 20 %, 40 % and 60 % (w/w), respectively (Table 1 a).

**Table 1:** Octenidine fatty acid coating of sutures with 40 cm in length and the resulting concentrations

		a) Coating solutions			b) Resulting antimicrobial suture preparation		
Coating type		Ratio of octenidine in fatty acid carrier	Drug weight (mg)	Fatty acid weight (mg)	Weight of octenidine (mg)	Weight of lauric or palmitic acid (mg)	Normalized drug weight (μg/cm)
octenidine-laurate	OL11	20 %	79.0	316.0	0.44	1.76	11
octenidine-palmitate	OP11						
octenidine-laurate	OL22	40 %	158.0	237.0	0.88	1.32	22
octenidine-palmitate	OP22						
octenidine-laurate	OL33	60 %	237.0	158.0	1.32	0.88	33
octenidine-palmitate	OP33						

**a)** Drug and fatty acid components were applied at given ratios above and dissolved in 10.0 ml ethanol to produce the specific coating solutions with 5 % mass (w/w). **b)** Octenidine content of coated sutures after preparation. The mean coating weight of 40 cm suture samples were determined at 2.2 ± 0.2 mg (*n* = 7). Weights on coated sutures for octenidine, fatty acid carrier and normalized mean drug weight per cm thread are given above

### Preparation of antimicrobial sutures

The sutures (40 cm in length, *n* = 7) were coated in a dipping process with the prepared antimicrobial coating solutions using a thermo-shaker (Heidolph Instruments GmbH, Germany) for 2 min at 35 °C at 150 rpm. Subsequently, sutures were fixed on a device and dried for 2 h (h) at room temperature. After this drying process, the coating weight was determined by using a precision balance (Atilon ATL-224; Acculab Inc., Massachusetts, USA). The drug amount normalized per length of sutures (μg/cm) was calculated for each drug concentration (20 %, 40 %, 60 %) via the measured coating weight (Table 1 b). Finally, coated sutures of 10 cm length were vacuum-sealed in sterile polyethylene bags and stored at room temperature. At the beginning of the experiments, coated sutures were cut into 1 cm, 2 cm and 3 cm long samples.

### Antimicrobial efficacy of coated sutures via agar diffusion test

Antimicrobial efficacy of sutures was tested via the agar diffusion test (*n* = 3) compared to Vicryl® Plus. According to CLSI criteria, suspensions of *Staphylococcus aureus* (ATCC® 49230™) were prepared to an optical density of 0.5 McFarland standard. Then, 1 ml of this suspension was plated uniformly on Mueller Hinton II Agar plates with 90 mm standard size. After removal of the supernatant and drying the petri dishes, suture samples were placed on the inoculated Agar plates and incubated at 37 °C overnight. After 24 h, zones of inhibitions were measured in millimeter (mm) by using a calliper perpendicular to the sutures. According to Ming et al. [10], this procedure was repeated daily by using the same suture samples for several days to recognize the remaining anti-bacterial activity until no detectable inhibition zone remained.

### Octenidine release from laurate and palmitate coatings

The octenidine release kinetics of the coated sutures were analysed over a period of 168 h in phosphate-buffered saline (PBS) at pH = 7.4. Sutures of 2 cm length (*n* = 6) were put in 1.5ml-cups (Eppendorf AG, Germany) with 1 ml PBS at 37 °C in a thermomixer MHR 23 (HLC-Biotech, Germany) at 200 rpm. Elution media was replaced by fresh PBS at fixed time intervals (after 0.5 h, 1.5 h, 3.5 h, 5.5 h, 7.5 h, 24 h, 48 h, 72 h, 96 h, 120 h, 144 h and 168 h). The release of octenidine was measured by absorption at a wavelength of 280 nm in a microplate photometer (Multiskan Go; Thermo Fisher Scientific GmbH, Germany). The amount of measured octenidine was normalized to the length of suture samples. Drug elution profiles were recorded by cumulating the released drug amounts over time. Ratios of the released octenidine were calculated relating to the drug content on suture samples at 168 h, depending on octenidine concentration of coated sutures.

### Biocompatibility study

In accordance with both the ISO 10993–5 guideline and the WST-1 assay instruction, analysis of *in vitro* cytotoxicity of coated sutures was performed by using mouse fibroblasts L-929 (ACC 2; DSMZ, Germany) and measuring the metabolic activity of cells in the presence of eluates from coated sutures. Cell cultures grew in the corresponding Dulbecco's Modified Eagle Medium (DMEM with 4.5 g/l D-glucose, Biochrom AG, Germany) consisting of 10 % fetal bovine serum at 37 °C and 5.0 % CO_2_ in a humidified atmosphere. Each well of a 96 well microtiter plate was filled with 10.000 cells and 200 μl DMEM, followed by incubation over 24 h. Eluates were generated simultaneously by placing coated sutures (*n *= 7) of 1 cm in length in 2 ml tubes containing 1.5 ml DMEM, for 24 h on a thermomixer at 37 °C and 300 rpm. Subsequently, cell culture’s media were replaced by the eluates after 24 h. After 48 h, each well was supplemented with 20 μl WST-1 reagent to commence cell reaction to generate formazan salts by cellular mitochondrial dehydrogenases. After a reaction time of 2 h at 37 °C, the amount of formazan salts was detected at 450 nm in an absorption reader. Metabolic activities were referred to L-929 cells used as growth reference without sutures. A threshold of 70 % according to ISO 10993–5 standard was used to claim “biocompatibility” for drug eluting sutures.

### Statistics

Statistical analysis was conducted by using the *student’s t-test* with significant level *p* < 0.05. Measurements based on mean values and standard deviations from at least three values. The Gaussian error propagation law was used to correct mean value calculations from several measurements.

## Results

### Reproducibility of antimicrobial suture coatings

Independently from the drug concentrations used, the mean coating weight - the difference between the uncoated and coated sutures of 40 cm in length was 2.2 mg ± 0.2 mg (*n* = 7). The amount of octenidine, fatty acid and the normalized amount of drug per length of thread was calculated for each drug concentration relative to this mean coating weight; 11, 22 and 33 μg/cm, respectively (Table 1). The weight of the triclosan containing Vicryl® Plus sutures was 2.7 μg/cm [28, 29], as declared by the manufacturer.

### Antimicrobial efficacy of coated sutures via agar diffusion test

**Fig. 1:**
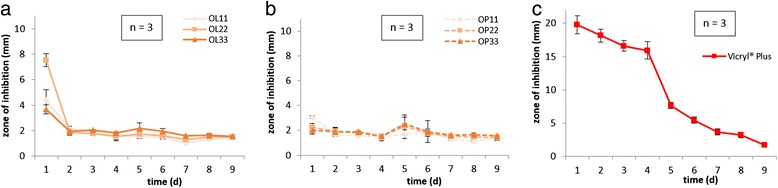
Antimicrobial long-term efficacies via agar diffusion tests using *S. aureus* lawns (2 × 10^8^ cfu/ml) showed inhibition zones over time for **a** octenidine-laurate coated sutures, **b** octenidine-palmitate coated sutures. Each coating type with three different octenidine concentrations 11, 22, and 33 μg/cm. **c** Vicryl® Plus as reference for commercial antimicrobial sutures

Lauric and palmitic coating of octenidine kept zones of inhibition relatively stable from the second to the ninth day of experiments from 1.9 mm to 1.6 mm at day 9 (Fig. 1). On the tenth day, experiments were discontinued because the threads lost stability by humidity on agar plates and could not be transferred anymore. Long-term protection was detected for the three drug concentrations, without depletion of the antimicrobial efficacy. Triclosan coated sutures (Vicryl® Plus) demonstrated zones from 19.8 mm to 1.7 mm of microbial inhibition for the same observation period (Fig. 1c).

### Octenidine release from laurate and palmitate coatings

**Fig. 2:**
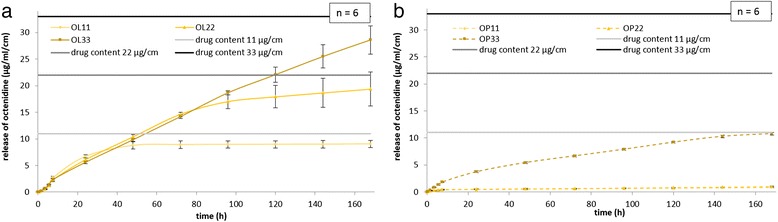
Elution profiles in PBS buffer at 37 °C from **a** octenidine-laurate coatings and **b** octenidine-palmitate coated PGA sutures. Elution profiles were determined for each coating type at 11, 22, and 33 μg/cm containing sutures. Horizontal lines depict the normalized drug contents per cm coated suture, representing the limit of drug release for each drug concentration

**Fig. 3:**
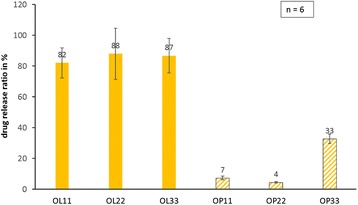
The percentage of drug release related to the drug content on coated sutures per cm length for each coating after 168 h of elution in PBS buffer

The release of octenidine from fatty acid coatings was measured over a period of 168 h via elution in PBS. As demonstrated, octenidine release (Fig. 2) depends on the used fatty acid as a coating component. Octenidine values cumulated over 168 h for octenidine-laurate coated sutures showed released concentrations of 9.0 μg/ml/cm (OL11), 19.4 μg/ml/cm (OL22), and 28.6 μg/ml/cm (OL33). Elution data with palmitate as retarding agent showed excellent delayed action. After 168 h, palmitate coated sutures reached 0.8 μg/ml/cm (OP11), 1.0 μg/ml/cm (OP22) and 10.8 μg/ml (OP33). The released amounts at 168 h were referred to the absolute amount of octenidine on coated sutures in percent (Fig. 3). The degree of octenidine release calculated for octenidine-laurate coatings results in 82 ± 10 % (OL11), 88 ± 17 % (OL22), and 87 ± 11 % (OL33). In comparison, octenidine-palmitate coatings released only 7 ± 1 % (OP11), 5 ± 1 % (OP22), and 33 ± 3 % (OP33) of the coated octenidine.

### Biocompatibility study

**Fig. 4:**
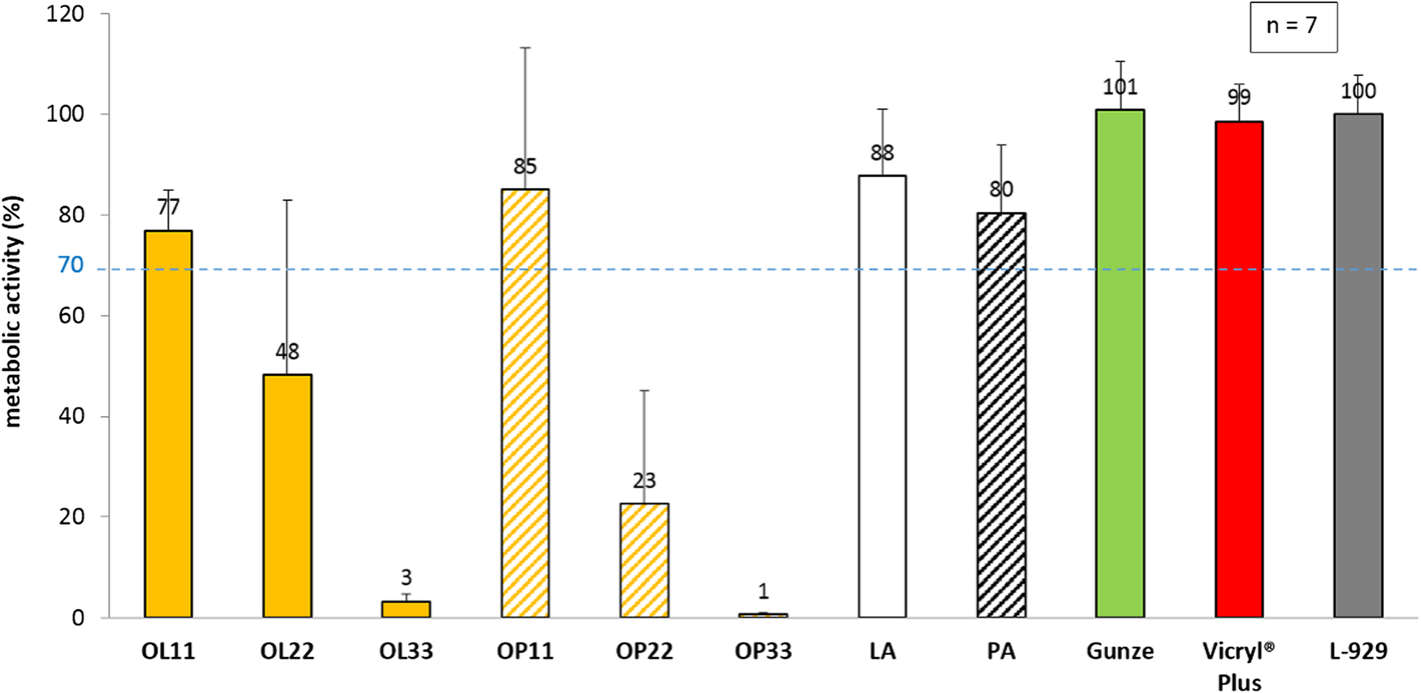
Metabolic cell activity of fibroblasts in the presence of eluates from octenidine coated sutures via WST-1 proliferation assay. L-929 mouse fibroblasts were incubated with suture eluates and used references: lauric acid (LA), palmitic acid (PA), uncoated sutures (Gunze), and Vicryl® Plus. All values were referred to cellular growth control, pure L-929 cells with culture media. Dashed line at 70 % pictures the level for acceptable lowering of metabolic activity according to ISO 10993–5:2009 in order to declare biocompatibility of medical devices

L-929 cells exposed to eluates from octenidine-laurate coatings showed metabolic activities of 77 ± 8 % (OL11), 48 ± 34 % (OL22), and 3 ± 1 % (OL33). Whereas, eluates from octenidine-palmitate resulted in metabolic activities of 85 ± 27 % (OP11), 23 ± 22 % (OP22), and 1 ± 0.3 % (OP33). The fatty acid coated references identified metabolic activities at 88 ± 13 % and 80 ± 13 % (lauric acid, palmitic acid). The control group with uncoated sutures reach a metabolic activity at 101 ± 10 % (Gunze) and Vicryl® Plus eluates demonstrated activities at 99 ± 7 % (Fig. 4).

## Discussion

Surgical site infections caused by sutures represent a serious problem in clinics. Antimicrobially coated sutures may reduce suture-associated surgical site infections. Bacterial contamination of the wound associated with sutures occurs through the exogenous pathway. Pathogens are introduced either during surgery or from natural skin flora via wicking effect. In the presence of suture materials a smaller amount of pathogens is often sufficient to cause an infection compared to wounds without foreign material [30]. In this study, we coated surgical sutures with 11, 22, or 33 μg octenidine per cm length either in a palmitate or laurate fatty acid as retarding carrier. The coated sutures were tested regarding their antimicrobial efficacy, octenidine release and biocompatibility. We found that the antimicrobial activity of octenidine coated sutures was as long as for triclosan coated sutures, like Vicryl® Plus. The favorable antimicrobial suture coating at 11 μg/cm was associated with high biocompatibility.

According to antimicrobial efficacy, inhibition zones of octenidine coated sutures on bacterial lawns were detected for up to 9 days. In comparison to the triclosan control group (Vicryl® Plus), the zones of inhibitions of octenidine were smaller, although the amount of octenidine on the sutures was significantly more than used with triclosan. This discrepancy in inhibition zones can be related to the presumably slower drug release by diffusion of octenidine over time compared to triclosan. This study was aimed to evaluate concentrations of octenidine on sutures in the balance of antimicrobial efficacy and biocompatibility. Released octenidine concentrations are thus low, but that may be desirable to protect the suture material, ensuring drug release for the first critical week of wound healing. Both octenidine- and triclosan coated sutures revealed a long lasting antimicrobial effect, but degradation of absorbable suture samples by hydrolysis caused termination of experiments after the ninth day. Despite this degradation process, no increase of inhibition zone could be detected as a possible indication of a sudden burst of octenidine release. Nevertheless, the results of this *in-vitro* model must be validated *in-vivo* prior to the application in humans, in order to dismiss concerns for acute toxicity as a consequence of the suture degradation process of octenidine coated sutures. The time dependency of inhibition zones did not differ greatly regarding the loaded drug concentrations of octenidine, so the three drug concentrations are equally effective absorbable sutures, as the essential protection is at least ensured for 9 days. Since the mechanism of wound healing provide an unaltered germ free de novo synthesis of tissue, this time period of antimicrobial protection should be sufficient to prevent infection during the first essential phase of wound healing.

The assessment of octenidine kinetics showed a slow continuous release over the first days of the experiment for each coated suture. The release of octenidine was delayed by the used fatty acid carriers and the drug’s low solubility in aqueous media. Triclosan coated sutures in such media, like Vicryl® Plus assessed similar durations of drug release [11]. Additionally, these data confirm our measured efficacy over at least 9 days for Vicryl® Plus sutures, considering the fact that our tested sutures from the EU containing less triclosan (max. 2.7 μg/cm), than Vicryl® Plus sutures available in the US (max. 4.7 μg/cm) [29]. Drug release kinetics over 7 days for octenidine coatings showed, that all type of coatings retarded the attached octenidine without full depletion. It is accepted that the antimicrobial coating remains between the suture filaments. Thus, the so-called wick effect of multifilament thread can be interrupted.

Referring to the drug carriers, palmitic acid in coatings showed a much slower drug release characteristic in comparison to lauric acid coatings demonstrating comparable antimicrobial efficacies. The ratio between released drug and loaded drug amounts on sutures after 7 days indicates that no coating type had fully washed-out the antimicrobial agent octenidine. Similar to our previous findings [25], we found that palmitic acid carriers delay the drug release more effectively than lauric acid carriers do. As anticipated, the octenidine release was dependent on the initial drug concentration in the suture coatings. Antimicrobial action could be demonstrated even for the lowest octenidine concentration (11 μg/cm) over 9 days. Especially, the palmitic acid coatings may guarantee a longer antimicrobial effect than lauric acid carriers may. Therefore, we recommend a combination of the drug octenidine with palmitic acid as carrier for long-term antiinfective protection of surgical sutures to avoid surgical site infections.

Biocompatibility, as defined by the ISO 10993–5:2009 guideline was reached for octenidine at the lowest concentration (11 μg/cm) with both coatings consisting of palmitic acid and lauric acid. Cytotoxicity tests of coated octenidine sutures showed a strong dose dependency. An increased drug concentration on loaded sutures, revealed an increased cytotoxic reaction. Octenidine-palmitate and -laurate sutures at 11 μg/cm showed similar metabolic activities in comparison to pure lauric and palmitic acid coated sutures. Compared to commercially available triclosan sutures (Vicryl® Plus), octenidine-palmitate coated sutures also met the ISO standard for biocompatibility.

The comparison of octenidine coated sutures to chlorhexidine coatings, tested in a previous study [25], showed different results in antimicrobial efficacy, drug release and biocompatibility by using the same antiseptic drug quantities. Octenidine sutures in lauric or palmitic acid showed initial slightly smaller inhibition zones than of chlorhexidine coatings during the first days of experiment. However, the duration of the inhibition zones lasted substantially longer for octenidine coatings, at least up to 9 days. Octenidine-laurate coated sutures compared to chlorhexidine-laurate coatings showed similar released drug amounts at 96 h, but the time span until flat drug kinetics were reached were much longer. In addition, the drug release of octenidine-palmitate sutures lasted longer and showed smaller released amounts at 96 h, referring to slower release compared to chlorhexidine-palmitate coatings. The reason for the longer drug release of octenidine can be related to the lower solubility of palmitic acid carrier and octenidine itself in aqueous media, such as PBS. Additionally, we observed that octenidine coatings were less cytotoxic by using the same drug doses compared to chlorhexidine coatings. Therefore, the drug release kinetics, antimicrobial activities and biocompatibility of octenidine coated sutures are superior compared to chlorhexidine coatings.

Our study has two main limitations: First, antimicrobial coated sutures were exposed to only one pathogenic strain, *S. aureus*. Herewith we wanted to primarily demonstrate the feasibility of effective triclosan alternatives. The activity of octenidine against other relevant bacteria as well as biofilm inactivation against *S. aureus* has been published [31]. Therefore, antimicrobial efficacy of coated sutures against other common pathogens of wound infections should be tested in the future. Second, the influence of the biofilm formation on the antimicrobial efficacy of the coated sutures was not simulated in our experiments. Regardless, the use of octenidine is likely to circumvent any of the acquired resistances which are limiting the extended use of triclosan.

## Conclusions

In the current study, novel octenidine coatings to render surgical sutures antimicrobial with three different concentrations based on palmitic/lauric acid were developed and analyzed. All novel coatings proved high long-term antimicrobial efficacy against *S. aureus*. Octenidine coatings with drug concentration of 11 μg/cm on sutures combine long-term antimicrobial efficacy up to 9 days and slow drug release with demonstrated high biocompatibility. The drug release was dependent on the fatty acid carrier and optimized delay was represented for palmitate. Compared to the commercially available Vicryl® Plus the antimicrobial efficacy of octenidine coated sutures was only slightly reduced. The potential disadvantages of triclosan are severe toxic side products such as dioxide and the promotion of multi drug resistance, which justifies the search for alternative substances for future use. However, despite these promising results, it needs to be clearly pointed out that it cannot be concluded from our findings that octenidine coated sutures are superior to the triclosan coated sutures. Therefore, further studies are necessary to prove that octenidine coated sutures represent a serious alternative to the currently commercially available triclosan coated sutures.

## Abbreviations

SSI: Surgical site infection
PGA: Polyglycolic acid
S. aureus: Staphylococcus aureus
USP: United States Pharmacopeia
OL: Octenidine in lauric acid coating
OP: Octenidine in palmitic acid coating
OL11: Octenidine-laurate coating at 11 μg/cm
OL22: Octenidine-laurate coating at 22 μg/cm
OL33: Octenidine-laurate coating at 33 μg/cm
OP11: Octenidine-palmitate coating at 11 μg/cm
OP22: Octenidine-palmitate coating at 22 μg/cm
OP33: Octenidine-palmitate coating at 33 μg/cm
rpm: Revolutions per minute
h: Hour
CLSI: Clinical and laboratory standards institute
mm: Millimeter
PBS: Phosphate-buffered saline
DMEM: Dulbecco’s modified eagle medium
EU: European Union
US: United States
